# Reliability of maximal isometric knee strength testing with modified hand-held dynamometry in patients awaiting total knee arthroplasty: useful in research and individual patient settings? A reliability study

**DOI:** 10.1186/1471-2474-12-249

**Published:** 2011-10-31

**Authors:** Ian FH Koblbauer, Yannick Lambrecht, Micheline LM van der Hulst, Camille Neeter, Raoul HH Engelbert, Rudolf W Poolman, Vanessa A Scholtes

**Affiliations:** 1 University of Applied Sciences, Amsterdam School of Health Professions, Amsterdam, The Netherlands; 2 Joint Research, Department of Orthopaedic Surgery, Onze Lieve Vrouwe Gasthuis, Amsterdam, The Netherlands; 3 Department of Rehabilitation, University Hospital Amsterdam (AMC), Amsterdam, The Netherlands

## Abstract

****Background**:**

Patients undergoing total knee arthroplasty (TKA) often experience strength deficits both pre- and post-operatively. As these deficits may have a direct impact on functional recovery, strength assessment should be performed in this patient population. For these assessments, reliable measurements should be used. This study aimed to determine the inter- and intrarater reliability of hand-held dynamometry (HHD) in measuring isometric knee strength in patients awaiting TKA.

****Methods**:**

To determine interrater reliability, 32 patients (81.3% female) were assessed by two examiners. Patients were assessed consecutively by both examiners on the same individual test dates. To determine intrarater reliability, a subgroup (n = 13) was again assessed by the examiners within four weeks of the initial testing procedure. Maximal isometric knee flexor and extensor strength were tested using a modified Citec hand-held dynamometer. Both the affected and unaffected knee were tested. Reliability was assessed using the Intraclass Correlation Coefficient (ICC). In addition, the Standard Error of Measurement (SEM) and the Smallest Detectable Difference (SDD) were used to determine reliability.

****Results**:**

In both the affected and unaffected knee, the inter- and intrarater reliability were good for knee flexors (ICC range 0.76-0.94) and excellent for knee extensors (ICC range 0.92-0.97). However, measurement error was high, displaying SDD ranges between 21.7% and 36.2% for interrater reliability and between 19.0% and 57.5% for intrarater reliability. Overall, measurement error was higher for the knee flexors than for the knee extensors.

****Conclusions**:**

Modified HHD appears to be a reliable strength measure, producing good to excellent ICC values for both inter- and intrarater reliability in a group of TKA patients. High SEM and SDD values, however, indicate high measurement error for individual measures. This study demonstrates that a modified HHD is appropriate to evaluate knee strength changes in TKA patient groups. However, it also demonstrates that modified HHD is not suitable to measure individual strength changes. The use of modified HHD is, therefore, not advised for use in a clinical setting.

## Background

Osteoarthritis (OA) is a major cause of disability throughout the world today [1]. Rising ageing populations, as well as advances in surgical procedures, suggest a future increase in the number of total knee arthroplasty (TKA) procedures performed in populations suffering from advanced OA [1]. As costs associated with disability amongst this patient population are significant [2], successful recovery from this surgical procedure is important for both the individual patient, and for the health care system as a whole. As therapeutic intervention and assessment findings in health care are currently aimed at utilizing best available evidence, progress in patient recovery should be evaluated using valid and reliable measurement methods [3]. By using valid and reliable measures in clinical practice, patient progress can be accurately evaluated and treatment protocols can be modified, thus increasing quality of patient care [3].

Patients awaiting TKA often experience pain, decreased range of motion (ROM), and decreased strength [4]. While pain and ROM are major focuses of evaluation in patients awaiting TKA, strength impairment should not be overlooked as it is linked to functional disability [5-8]. According to the International Classification of Function, Disability, and Health (ICF), muscle strength (muscle power functions) is considered to be a category of major importance in OA, being included in the Brief ICF Core Set [9]. Routine assessment of muscle function during patient examination should, therefore, be implemented in clinical orthopedic practice [10].

Current methods of strength testing include Manual Muscle Testing (MMT), isokinetic dynamometry, and hand-held dynamometry (HHD). Although MMT is a practical option for strength testing, its questionable reliability and subjective nature in obtaining strength measures has created an interest in alternative methods of strength testing [11].

The use of isokinetic dynamometry for measurement has been shown to be highly reliable and is currently the golden standard for strength measures [12]. Velocity of strength development, which has been linked to functional recovery [13], can be measured with these devices. However, considerable costs and low accessibility to these devices make them uncommon in a clinical setting [12].

HHD is a relatively inexpensive and portable device, making it a practical alternative to isokinetic dynamometry [10]. HHD has been shown to be reliable for evaluation of knee strength in various populations including patients with cancer [14], pediatric patients [15,16], geriatric patients [17], patients with chronic obstructive pulmonary disease (COPD) [18], and patients with dementia [5]. A recent review stated that isometric muscle testing using HHD is reliable, and should be integrated into routine clinical examination of orthopedic hip and knee patients [10]. However, no studies including patients undergoing TKA were included in this review [10]. A study by Kwoh et al. evaluated unaffected knee strength using HHD in patients awaiting TKA [19] and another by Gagnon et al. evaluated knee strength in patients recovering from TKA using a chair-fixed HHD [6]. Additional studies have been performed in patients with OA [20-22], however, TKA patients differ from OA patients in that they have advanced osteoarthritic changes, which may affect muscle function [23]. This current lack of literature indicates the need to evaluate the inter- and intrarater reliability of isometric strength measures using HHD in patients awaiting TKA.

To determine reliability, the intraclass correlation coefficient (ICC) is a commonly used statistical measure [24]. A disadvantage of the ICC, however, is that the statistic is dependent on the variance of measures within the study population. An increased reliability coefficient as a result of increased between-subject variance can, therefore, be misleading. Furthermore, the clinical applicability of the ICC is minimal, as it provides an index of reliability for group, but not individual measures. Thus, in addition to the ICC, it is important to use statistical measures such as the Standard Error of Measurement (SEM) and the Smallest Detectable Difference (SDD) to evaluate reliability [25]. The SDD, derived from the SEM, represents measurement error and, therefore, the threshold that must be overcome to ensure real change. As the SDD represents a number using the same units as the original measure, this number has considerable value for clinical use.

Previous literature evaluating reliability of HHD has demonstrated high reliability, in terms of ICC values, and relatively low measurement error, in terms of SEM and SDD values [6,14,22]. Based on these results, the hypothesis that HHD would yield high reliability and low measurement error in both the affected and unaffected knee pre-operatively was formulated. Therefore, the aim of the study was to assess the inter- and intrarater reliability of isometric strength measures using HHD in patients awaiting TKA. Moreover, the study aimed to illustrate that the clinical applicability of the ICC is minimal, and results should, therefore, be appropriately presented with more clinically applicable measures such as the SEM and SDD.

## Methods

### Design

The study evaluated inter- and intrarater reliability of HHD isometric strength testing in patients awaiting TKA. Two examiners, A and B, were two physical therapy students from the University of Applied Sciences, Amsterdam School of Health Professions, and performed all measurements. The strength testing protocol was developed by the two examiners together with two physical therapists and one researcher experienced in both clinical and research-based use of HHD.

### Subjects

The study sample included patients awaiting TKA who had a minimum of one appointment in the hospital within a four-week period. Minimum age of 18 years and adequate knowledge of Dutch or English were required to take part in the study. Patients were excluded if they were unable to flex their knee to 90°, if pain interfered with the testing procedure, or if additional disorders influenced the patient's musculoskeletal system. The study was given ethical approval by the institutional medical ethical review board (Verenigde Commissies Mensge-bonden Onderzoek [VCMO], Nieuwegein) from the Onze Lieve Vrouwe Gasthuis hospital.

**Figure 1:**
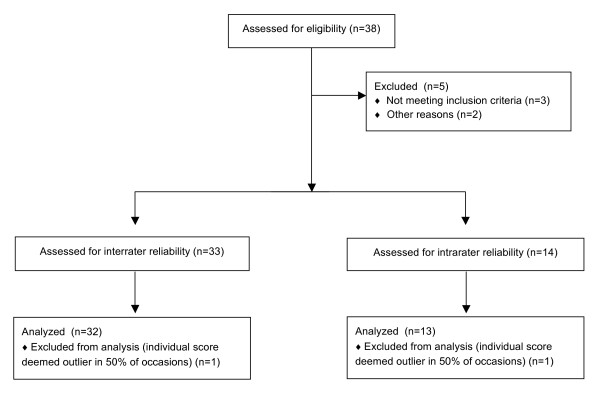
**Flow diagram**: Process of exclusion and number of patients per inter- and intrarater reliability.

As illustrated in Figure 1, a total of 38 patients were invited to participate following the pilot phase. Due to scheduling difficulties, patient language barriers, and ROM limitations, five patients were excluded. The remaining 33 patients provided written informed consent and participated in the study. Fourteen patients volunteered to undergo additional testing on a second date to evaluate intrarater reliability. Subjects were tested on dates and times corresponding with other appointments in the hospital.

### Anthropometric characteristics

All patient demographics (date of birth, weight, height) were retrieved from the Hospital Information System (ZIS - X/Care - McKesson Nederland B.V.). All personally identifiable information was made anonymous when placed in the database.

### Equipment

Isometric muscle strength was quantified using a Citec hand-held dynamometer (type CT 3001, C.I.T. Technics). The HHD was calibrated as per the manufacturer's specifications prior to testing each patient. The Citec has a test range of 0 to 500 N according to the manufacturer.

### Testing procedure

#### Protocol

**Figure 2:**
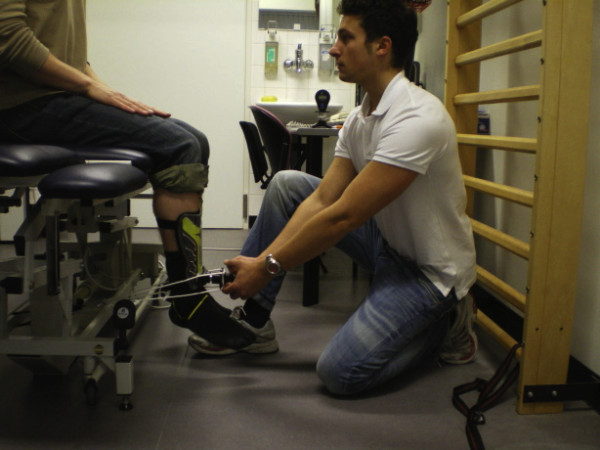
**Clinical assessment of muscular isometric knee extension strength using a modified hand-held dynamometer**.

**Figure 3:**
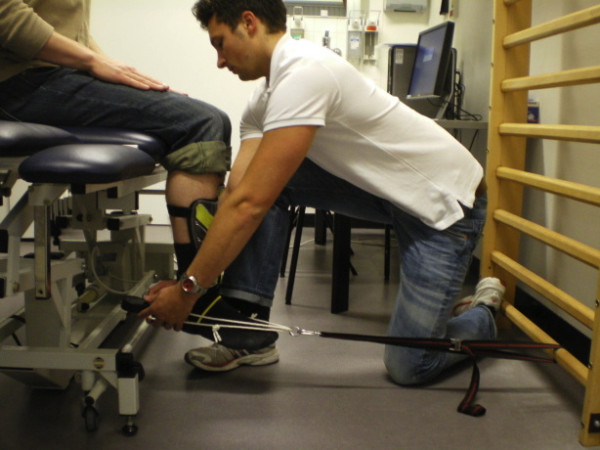
**Clinical assessment of muscular isometric knee flexion strength using a modified hand-held dynamometer**.

Testing was performed with the patient seated on a Huntleigh-Akron treatment table (Huntleigh Akron Ltd). Patients' hips and knees were positioned at 90°. The patient was instructed to remain seated in an upright position and place both hands on his or her upper legs to avoid compensation. The "make" method for strength testing was performed rather than the "break" method as it has been shown to have better reliability and provide more accurate measures [26,27]. As the pilot study revealed that some TKA patients were, indeed, too strong to allow the examiners to withstand the forces necessary to accurately perform the "make" test, the HHD was modified with straps to provide support in holding the HHD during testing (see Figure 2 &3). Straps were fixated to standardized attachments on the treatment table for extension and the wall ladder for flexion. The position of the treatment table was standardized in relation to the wall and floor. The length of the straps allowed for an isometric contraction to be performed with the knee at 90° during both flexion and extension. As discrepancies between patients' knee angles were minimal using standardized strap lengths, length adjustments between patients were not necessary.

For extension, the HHD was positioned perpendicular to the anterior aspect of the tibia, 5 cm proximal of the medial malleolus, as described in previous studies [7,28]. Shin guards were placed on the patient's lower legs to allow for both standardization of the HHD placement and pain reduction from the pressure created by the HHD. For flexion, the HHD was positioned on the posterior aspect of the calcaneus.

#### Strength testing

Prior to testing, the patient was given a visual analogue scale (VAS) and was asked to place a line corresponding to the severity of pain in both the affected and unaffected knee. The corresponding number (zero representing no pain and 10 representing unbearable pain) was then recorded by each examiner for both trials. The patient was told to inform the examiner of any significant pain or general discomfort during the testing procedure, and was ensured that the testing procedure could be stopped at any time upon request. Each patient performed four strength measures per leg for both flexion and extension, the first measure being used as a familiarization trial. For analysis, the mean maximal strength of the 2^nd^, 3^rd^, and 4^th ^measures were calculated and corrected for bodyweight. All knee extension strength measures were first taken, followed by flexion. The initial measurement was performed on the unaffected leg, followed by the affected leg, and thereafter alternated in a similar fashion for both flexion and extension. Thirty seconds of rest was taken between strength measures of each leg to allow for muscle recovery [18,29]. Examiner A performed the first test procedure. Examiner B then performed the same testing procedure after a three-minute rest period to allow for complete muscle recovery [30]. The patient was instructed to gradually build up strength for two seconds to avoid explosive contraction, then to continue with a three-second maximal contraction as used in previous studies [28,31]. Standardized encouragement for maximal contraction was verbally provided ("GO, GO, GO") by the examiner for the three-second period. Each tester was blinded regarding inter- and intrarater strength measures obtained.

### Statistical Methods

Patient demographics and strength measures were analyzed using Statistical Package for Social Sciences for Windows (SPSS 17.0, SPSS Inc). Differences in pain were analysed with unpaired and paired Student T-tests, using a Bonferroni correction for multiple tests. Inter- and intrarater reliability was examined by means of the generalizability theory [25]. The intraclass correlation coefficient (ICC) was used to calculated inter- and intrarater reliability for strength measurements using the formula: Var_Subject_/(Var_Subject _+ Var_Occasion _+ Var_Error_). A twoway random effects model (ICC_2,1_) for an average of three trials was used. According to Portney and Watkins [32] ICCs above 0.75 indicate good reliability. Utilizing data from SPSS, SEM and SDD were calculated as: √(Var_Error_. + Var_Occasion_) and √(2)*1.96*SEM, respectively [25]. Lower SEM and SDD values indicate lower measurement error, and thus better reliability. All SEM and SDD values are additionally presented as a percentage of the mean maximal strength.

#### Outliers

Subject strength variation was evaluated using a box-plot. Interrater strength scores of flexion and extension in both knees were analyzed. Individual scores deemed outliers in 50% of occasions were excluded from the study sample.

## Results

Of the 33 patients involved in the final study, one male patient was identified as an outlier and excluded. Of the remaining 32 patients, 26 subjects (81.3%) were female and six subjects (18.8%) were male. Interrater reliability was evaluated in all 32 patients. In a subgroup of 13 patients, intrarater reliability was evaluated. The mean time interval between the two testing dates was 11 days (SD 2; range 2-27). Patient characteristics and pain scores are summarized in Table 1. Patients tended to experience more pain with examiner A than with examiner B, although this was not significant. No patients had to stop with testing due to excessive pain and no significant associations between pain scores and strength measures were found.

**Table 1:** Patient Characteristics and Pain Scores

		All patients (n = 32)		Subgroup (n = 13)		
		**Frequency (%)**	**Mean (SD)**	**Frequency (%)**	**Mean (SD)**	

Gender						
Female		26 (81.3)		11 (84.6)		
Male		6 (18.8)		2 (15.4)		
Knee to be operated						
Right		13 (40.6)		5 (38.5)		
Left		18 (56.3)		7 (53.8)		
Both		1 (3.1)		1 (7.7)		
Age (years)			68.38 (9.66)		67.54 (10.20)	
Height (centimetres)			163.94 (18.03)		167.23 (11.13)	
Weight (kilograms)			81.27 (13.25)		81.91 (14.15)	
BMI			29.13 (4.15)		29.30 (4.51)	

Pain (VAS 0-10)					*Day 1*	*Day 2*
*A *	Affected knee		2.6 (2.4)		2.89 (2.92)	2.96 (2.73)
	Unaffected knee		1.5 (1.9)		1.49 (2.00)	1.23 (1.58)
*B *	Affected knee		1.8 (2.3)		1.63 (2.12)	2.12 (2.71)
	Unaffected knee		0.6 (1.1)		0.49 (1.03)	0.53 (1.27)

SD: standard deviation, BMI: Body Mass Index, VAS: Visual Analogue Scale, A: examiner A, B: examiner B

ICC, SEM, and SDD values for inter- and intrarater reliability of HHD strength measures are presented in Table 2.

**Table 2:** ICC, SEM, and SDD values for inter- and intrarater reliability of HHD strength measures.

INTERRATER (n = 32)	Strength		Reliability		
	
	Mean (SD) *Examiner A*	Mean (SD) *Examiner B*	ICC (95% CI)	SEM*	SDD*
*Extension*					
Affected	2.73 (1.03)	2.62 (0.95)	0.96 (0.91 - 0.98)	0.21 (7.8%)	0.58 (21.7%)
Unaffected	3.14 (1.04)	2.97 (1.08)	0.95 (0.85 - 0.98)	0.25 (8.0%)	0.68 (22.3%)
*Flexion*					
Affected	1.01 (0.43)	1.04 (0.43)	0.90 (0.81 - 0.95)	0.13 (13.1%)	0.37 (36.2%)
Unaffected	1.04 (0.47)	1.04 (0.46)	0.94 (0.88 - 0.97)	0.11 (10.8%)	0.31 (29.8%)

**INTRARATER (n = 13)**	**Strength**		**Reliability**		
	
	**Mean (SD) ** ***Day 1***	**Mean (SD) ** ***Day 2***	**ICC (95% CI)**	SEM**	SDD**

**Examiner A**					
*Extension*					
Affected	2.73 (1.03)	2.61 (1.04)	0.92 (0.74 - 0.97)	0.30 (11.3%)	0.84 (31.4%)
Unaffected	3.14 (1.04)	2.70 (0.92)	0.94 (0.83 - 0.98)	0.24 (8.1%)	0.66 (22.4%)
*Flexion*					
Affected	1.01 (0.43)	0.85 (0.38)	0.76 (0.36 - 0.92)	0.19 (20.2%)	0.52 (55.9%)
Unaffected	1.04 (0.47)	0.88 (0.35)	0.79 (0.45 - 0.93)	0.20 (20.7%)	0.55 (57.5%)

**Examiner B**					
*Extension*					
Affected	2.62 (0.95)	2.49 (0.99)	0.97 (0.90 - 0.99)	0.18 (6.8%)	0.48 (19.0%)
Unaffected	2.97 (1.08)	2.73 (1.02)	0.93 (0.69 - 0.98)	0.26 (9.3%)	0.73 (25.6%)
*Flexion*					
Affected	1.04 (0.43)	0.91 (0.38)	0.80 (0.47 - 0.94)	0.16 (16.0%)	0.43 (44.4%)
Unaffected	1.04 (0.46)	0.95 (0.44)	0.90 (0.69 - 0.97)	0.14 (14.4%)	0.40 (39.8%)

Mean, SD, SEM and SDD values are presented in N/kg.

* Percentages of mean maximal strength measures of Examiner A and B

** Percentages of mean maximal strength measures of Day 1 and Day 2

N/kg: Newtons per kilogram, SD: Standard deviation, ICC: Intraclass correlation, 95% CI: 95% Confidence interval, SEM: Standard error of measure, SDD: Smallest detectable difference, SEM = √(occasion variance + error variance), SDD = √(2)*1.96*SEM

Interrater ICC scores were good, ranging from 0.90 to 0.96 for all measures. Interrater SEM values ranged from 7.8% to 13.1% of mean maximal strength, whereas SDD values ranged from 21.7% to 36.2%.

To clarify the results of the SEM and SDD, the results for the interrater reliability, affected knee extension (see Table 2), will be described in more detail. For knee extension, the SEM was 7.8% (0.21 N/kg). This means individual scores had a measurement error of 7.8% on average. The SDD was 21.7% (0.58 N/kg). For clinical application this means that, for an individual, only strength changes as large as 21.7% can be interpreted as a real change with 95% confidence.

The overall intrarater ICC values ranged between 0.76 to 0.97. These values were lower than interrater ICC values, but still considered to have good reliability. SEM values ranged from 6.8% to 20.7% of mean maximal strength, whereas SDD values ranged from 19.0% to as high as 57.5%.

Both inter- and intrarater reliability were systematically higher for extension than for flexion, as displayed by higher ICC and lower SEM and SDD values. No systematic differences were found between the affected and unaffected knee.

## Discussion

In this reliability study, the formulated hypothesis was confirmed with good to excellent ICC values found for both inter- and intrarater reliability, indicating good to excellent reliability. Contradictory to the hypothesis, SEM and SDD values indicated high measurement error, illustrating the limited usefulness of HHD to evaluate individual patients in a clinical setting.

Performing a pilot study to evaluate practicality and provide examiner training strengthened the study. Regarding participants, the study had an adequate sample size when compared with other literature evaluating the use of HHD in patients undergoing TKA [6,19]. Additionally, all eligible patients participated in the study, providing a good representation of the patient population involved. Regarding the testing procedure, a rigidly standardized protocol was used to ensure minimal error. Modifications made to assist holding the HHD eliminated factors such as inadequate examiner strength and excessive patient strength, which have been shown to be limitations in past studies [6,14]. Results were, therefore, not dependent on either examiner or patient strength and can consequently be applied to a general clinical setting. The modification made to the HHD, hereafter referred to as "modified HHD", was also not complex as it was simply fixated using straps. A level of practicality was still maintained, making the protocol useful in a clinical setting when compared to more elaborate modifications made in past studies [6,31].

The high ICC values for both inter- and intrarater reliability indicate that HHD strength measurements were reliable. This finding is comparable with other studies evaluating HHD [6,7,14,18,19,22,33,34]. A drawback of this interpretation of reliability, however, is that the ICC interprets group measures rather than individual measures. This raises concerns and makes previous conclusions that HHD is appropriate for routine clinical examination [10] questionable.

When analyzing this study's reliability using the SEM and SDD, high measurement error was indicated. Previous studies evaluating HHD, in contrast, have demonstrated only low to moderate measurement error in terms of SEM and SDD [14,22]. These studies, however, were performed on other populations and used non-modified HHD [14,22]. Results of the current study would, therefore, be more comparable with a study by Gagnon et al., where strength testing was performed using a modified HHD in patients recovering from TKA [6]. While Gagnon et al. did find lower measurement error in terms of SEM values (2.9-9.9% of mean strength), a chair fixed dynamometer was used in this study [6], which closely represented an isokinetic dynamometer. This may be an explanation for the discrepancy between findings as the modified HHD used in the current study was, most likely, not held as stably as the chair fixed dynamometer. In contrast to Gagnon et al.'s findings [6], the high SEM and SDD values found in this study indicate high measurement error.

Regarding clinical relevance, while the ICC values indicate that the use of a modified HHD is appropriate to measure groups for clinical trials, its use in a clinical setting must further be evaluated with the SDD. As the SDD ranged from 19% to 31% for extension and 30% to 58% for flexion, a strength gain of as high as 31% for extension and 58% for flexion would be necessary to detect real change in strength. The clinical usefulness of these measures must, therefore, be explored. As factors such as swelling and pain can play a role in limiting strength directly following operation [35], intervals of four weeks, six months, and one year have been used for strength assessment in TKA patients [35-37]. Knee extensor strength measures taken approximately four weeks post-operatively have shown strength losses of approximately 60% in patients recovering from TKA [36,37]. Strength measures at three months and one year post-operatively have shown losses of 34% and 13%, respectively [37]. Knee flexor strength measures, often neglected, have shown decreases of only 17% three to six months following TKA [38]. Given these numbers, strength measures using HHD for this patient population appears to be of limited value. While SDD's as high as 58% for flexion are too high to be of clinical use, SDD's as high as 31% for extension would generally be suitable only for long-term strength increases experienced by a patient following TKA. A typical patient could, therefore, only be evaluated for strength recovery after several months since previous strength gains would not accurately be detected.

Contributing factors to high measurement error should be explored. Firstly, standardized times of testing were not possible due to patient scheduling. This lack of standardization is a factor which may have had an influence on all measures. Secondly, interrater reliability was generally better than intrarater reliability for ICC, SEM, and SDD. This outcome was not expected as similar studies evaluating HHD have reported opposite findings [6,7,14]. For this study, the number of subjects involved for analysis of reliability may have contributed to this finding. As there were almost twice as many inter- (n = 32) than intrarater reliability (n = 13) patients, these numbers may have had an effect on reliability. Additionally, the study evaluated the intrarater reliability of strength measures over a relatively longer period of time (within one month) when compared with other literature [7,14]. Therefore, the patients' variation over this time period may have affected intrarater reliability. Another contributing factor for the unexpected reliability distribution may have been the dates of testing. All intrarater reliability patients performed their 2^nd ^testing procedure closer to their date of operation. Patients awaiting surgery have been shown to experience emotional change as their operation day nears [39]. This emotional change may have had an effect on the patients' performance during testing.

Thirdly, when analyzing reliability of flexion and extension, it was found that extension (SDD = 19-31%) was more reliable than flexion (SDD = 30-58%). Previous studies have described difficulties in isolating the knee flexors during testing, resulting in hip flexion [28]. This may have had an influence on force exerted on the HHD, therefore affecting strength measures [28]. As fixation points of the HHD support straps were different for flexion and extension, differing lever arms created as a result of the strap placement may have also influenced measures. As the HHD remained supported by the strength of the examiners in addition to the support provided by the straps, this potential source of error was, however, minimized. Another possible explanation involves random error, which occurred in all measures, exerting much more of an effect on the lower flexion scores. The proportional difference between error values and measurement values in flexion and extension would, therefore, result in much higher error for flexion measures.

Suggestions for further research include utilizing a similarly standardized protocol to establish the reliability of HHD in a healthy population homogeneous to the current sample in terms of strength and age. Comparing the reliability of the modified HHD to a standard HHD would additionally be beneficial to suggest one method over the other. Analysis of these results may provide a threshold in Newtons to establish the clinical setting and patient population in which the use of a standard HHD is still appropriate. Results could additionally be compared with measures taken with an isokinetic dynamometer to determine accuracy of these measures. Intrarater reliability analysis may also be evaluated using shorter test intervals, therefore minimizing patient variance and better determining true measurement error. Furthermore, relations between HHD strength measures and functional measures should be evaluated to determine whether HHD strength measurement can, indeed, provide a measure on which to gauge functional recovery.

## Conclusion

Modified HHD strength measures produced good to excellent ICC values for both inter- and intrarater reliability. The use of modified HHD is, therefore, appropriate for evaluating isometric knee strength measures in patient groups undergoing TKA. High SEM and SDD values, however, indicated high measurement error for individual measures. The use of modified HHD is, therefore, not advised for use in a clinical setting to evaluate strength in individual patients undergoing TKA.

## List of abbreviations

TKA: Total knee arthroplasty; ROM: Range of motion; MMT: Manual muscle testing; HHD: Hand-held dynamometry; COPD: Chronic obstructive pulmonary disease; OA: Osteoarthritis; ICC: Intraclass correlation coefficient; SEM: Standard error of measure; SDD: Smallest detectable difference; N: Newtons; BMI: Body mass index; VAS: Visual analogue scale.

## Competing interests

The authors declare that they have no competing interests.

## Authors' contributions

Study design: IFHK, YL, MLMH, CN, RHHE, RWP, VAS; Subject measures: IFHK, YL; Analysis and interpretation of data: IFHK, YL, MLMH, CN, VAS; Manuscript preparation: IFHK, YL, CN, RHHE, RWP, VAS; Statistical analysis: IFHK, YL, VAS. All authors have reviewed and approved the manuscript.

## Pre-publication history

The pre-publication history for this paper can be accessed here:

http://www.biomedcentral.com/1471-2474/12/249/prepub
